# Proactive Measures to Combat a SARS-CoV-2 Transmission Among High Risk Patients and Health Care Workers in an Outpatient Dialysis Facility

**DOI:** 10.3389/fphar.2020.600364

**Published:** 2021-03-23

**Authors:** Jayandiran Pillai, Pagollang Motloba, Keolebogile Shirley Caroline Motaung, Carole Wallis, Lovelyn Uzoma Ozougwu, Debashis Basu

**Affiliations:** ^1^ Department of Surgery, University of the Witwatersrand, Johannesburg, South Africa; ^2^ Department of Community Dentistry, Sefako Makgatho Health Sciences University, Pretoria, South Africa; ^3^ Department of Technology Transfer and Innovation, Durban University of Technology, Durban, South Africa; ^4^ BARC-SA and Lancet Laboratories, Johannesburg, South Africa; ^5^ Department of Public Health Medicine, Steve Biko Academic Hospital and University of Pretoria, Pretoria, South Africa; ^6^ WHO Collaborating Centre for and Social Determinants of Health and Health in All Policies, Pretoria, South Africa

**Keywords:** COVID-19, biomarkers, SARS-CoV-2, RT-PCR, renal dialysis

## Abstract

**Background:** End-stage-renal-failure (ESRF) patients attending clustered out-patient dialysis are susceptible to SARS-CoV-2 infection. Comorbidities render them vulnerable to severe COVID-19. Although preventative and mitigation strategies are recommended, the effect of these are unknown. A period of “potential-high-infectivity” results if a health-care-worker (HCWs) or a patient becomes infected.

**Aim:** We describe and analyze early, universal SARS-CoV-2 real time reverse transcription polymerase chain reaction (RT-PCR) tests, biomarker monitoring and SARS-CoV-2 preventative strategies, in a single dialysis center, after a positive patient was identified.

**Methodology:** The setting was a single outpatient dialysis center in Johannesburg, South Africa which had already implemented preventative strategies. We describe the management of 57 patients and 11 HCWs, after one of the patients tested positive for SARS-CoV-2. All individuals were subjected to RT-PCR tests and biomarkers (Neutrophil-Lymphocyte Ratio, C-reactive protein, and D-Dimer) within 72 h (initial-tests). Individuals with initial negative RT-PCR and abnormal biomarkers (one or more) were subjected to repeat RT-PCR and biomarkers (retest subgroup) during the second week. Additional stringent measures (awareness of viral transmission, dialysis distancing and screening) were implemented during the period of “potential high infectivity.” The patient retest subgroup also underwent clustered dialysis until retest results became available.

**Results:** A second positive-patient was identified as a result of early universal RT-PCR tests. In the two positive-patients, biomarker improvement coincided with RT-PCR negative tests. We identified 13 individuals for retesting. None of these retested individuals tested positive for SARS-CoV-2 and there was no deterioration in median biomarker values between initial and retests. Collectively, none of the negative individuals developed COVID-19 symptoms during the period “potential high infectivity.”

**Conclusion:** A SARS-CoV-2 outbreak may necessitate additional proactive steps to counteract spread of infection. This includes early universal RT-PCR testing and creating further awareness of the risk of transmission and modifying preventative strategies. Abnormal biomarkers may be poorly predictive of SARS-CoV-2 infection in ESRF patients due to underlying illnesses. Observing dynamic changes in biomarkers in RT-PCR positive and negative-patients may provide insights into general state of health.

## Introduction

The COVID-19 pandemic (causative agent: SARS-CoV-2) has been unrelenting since the first case was reported in Wuhan in December 2019 (WHO, 2020a). In South Africa the number of cases has increased exponentially (5,647 cases as on April 30, 2020 in comparison to 725,452 cases on October 31, 2020) (South Africa National Department of Health, 2020). The clinical management of cases was also changed during this period due to better understanding of the course of the disease (National Institute for Communicable Diseases, 2020). Risk factors for severe SARS-CoV-2 infection (COVID-19) include cardiovascular disease, diabetes, hypertension, older age, chronic lung disease and cancer (WHO, 2020b). It is reported that 15–20% of infected individuals may progress toward severe disease (interstitial pneumonia, acute respiratory distress syndrome and systemic inflammation) (Jordan et al., 2020).

Patients who suffer from haemodialysis dependent, End Stage Renal Failure (ESRF) are a specific high-risk group who are susceptible to SARS-CoV-2 infection (Chen et al., 2020a). The multiple comorbidities in ESRF patients may infer rapid COVID-19 progression and death. These patients attend, on average, three hourly dialysis sessions several times a week, during which they are in close proximity to each other and to health care workers (HCW). This is a life-long commitment. The preferred distance between dialysis stations is 900 mm (South Africa National Department of Health, 2013). Satellite community units (not linked to hospitals) may be further hampered by space. Out-patient dialysis is associated with clustering during transport, pre-dialysis seating, hours of dialysis sessions and staff interaction. In the event of SARS-CoV-2 exposure, dialysis shut down, rearrangement, reallocation of patients and ensuring isolated outpatient dialysis is practically impossible. The EUDIAL Working Group provided routine, broad recommendations for SARS-CoV-2 prevention, mitigation and containment in dialysis centers (Basile et al., 2020). However, recommendations in the event of one or more individuals being infected, are unclear and have not been assessed (Corbett et al., 2020). The detection of SARS-CoV-2 infection in a dialysis unit patient, is likely to result in a period of ‘potential high infectivity’ within the Unit. This infective risk may span over two weeks when one considers viral incubation and latency (WHO, 2020c).

Non-specific acute phase reactants have been reported as biomarkers to predict acute and severe COVID-19. Trends in biomarkers in hospitalized patients are used to monitor severity of disease (Kermali et al., 2020; Ulhaq and Soraya, 2020; Terpos et al., 2020; Lan et al., 2020).

Reports have also suggested that laboratory parameters may predict a SARS-CoV-2 positive test (LABMATE, 2020; Mardani et al., 2020; Ferrari et al., 2020). A high incidence of early lymphopenia and high CRP were noted in COVID-19 ESRF patients (Aydin Bahat et al., 2020; Goicoechea et al., 2020). However, similar biomarkers are used to monitor and to diagnose bacterial infections and thrombotic risk in ESRF patients (Cruz et al., 2011; Nakazato et al., 2015; Gubensek et al., 2016).

It is currently unclear how these biomarkers may be used to potentially diagnose SARS-CoV-2 and monitor ESRF patients in the event of a SARS-CoV-2 outbreak. The aim of the study is to describe and analyze early, universal SARS-CoV-2 real time reverse transcription polymerase chain reaction (RT-PCR) tests, biomarker monitoring and SARS-CoV-2 preventative strategies, in a single dialysis center, after a positive patient was identified.

## Methodology

In this observational, analytical case study, we analyzed the potential benefits of early SARS-CoV-2 RT-PCR testing, biomarker monitoring and SARS-CoV-2 preventative strategies during a period of “*potential high infectivity*.” We also analyze biomarker trends to identify patients with potential SARS-CoV-2 false negative results.

The setting was an outpatient dialysis facility in Johannesburg, South Africa. The 400 sq. meter Unit is divided into four dialysis sections, a communal “waiting area,” a worker’s station and an isolation cubicle. The dialysis sections are open areas with a dialysis distance of 1 m apart. Each section may accommodate up to eight patients. There are three dialysis sessions held on a Mondays, Wednesdays and Fridays and two sessions on Tuesdays, Thursdays and Saturdays, with 20 dialysis machines servicing the Unit. Each patient attends either two or three dialysis sessions per week depending on individual renal function profile as assessed by a nephrologist. Although HCWs were assigned to specific stations they assisted at other stations when needed. Five of the HCWs worked at other dialysis units. Preventative strategies (COVID-19 education, skin temperature monitoring, hand sanitizing, routine face mask use, dialysis and social distancing and symptomatic screening on entry) had already been implemented for a month prior to identification of the positive patient. Physical distancing was initiated in the waiting area by rearranging chair distances to 2 m apart. Symptom screening was implemented in the waiting area by handing out a standard questionnaire to each patient. The COVID-19 symptoms in the questionnaire included cough, fever, sore throat, shortness of breath, diarrhea and vomiting. A specific comment was requested regarding exacerbation of symptoms and appearance of new symptoms. Other details in the questionnaire included contact with SARS-CoV-2 positive individuals and use of public transport. Each patient had their skin temperature measured prior to dialysis. Similar data and temperature monitoring was requested for all HCWs. All individuals were advised to avoid the use of public transport if possible. The Unit was considering a random RT-PCR testing policy which had not as yet been implemented. None of the patients nor HCWs reported symptoms in the preceding week and none had SARS-CoV-2 real time reverse transcription polymerase chain reaction tests (RT-PCR) performed in the past. All 68 individuals were considered to be at risk for SARS-CoV-2 infection.

On the April 22, 2020, a 35-year-old male tested positive for SARS-CoV-2, in an outpatient haemodialysis facility in Johannesburg, South Africa (routine investigation prior to urgent surgery). There were no symptoms nor signs suggestive of COVID-19 and he attended usual dialysis the week before.

After identifying the Index case all patients and HCWs, were alerted telephonically and advised to self-isolate at home. Preventative policies, COVID-19 symptom awareness and social distance policies were reinforced telephonically. Contact tracing was undertaken by the infection control unit of the hospital linked to the dialysis Unit. This was done in collaboration with the Provincial department of Health based on government policy.

A pathology laboratory was notified of the risk within the Unit. Laboratory staff who were trained in SARS-CoV-2 preventative strategies were sent to the dialysis Unit for sample collection. The following tests were requested in all 68 individuals within 72 hours: RT-PCR, full blood count (FBC) including Neutrophil-Lymphocyte Ratio (NLR), C-reactive protein (CRP) and D-Dimer (Initial tests). These tests were specifically requested for the study and funded by the dialysis Unit. Initial tests were conducted at the dialysis Unit in maximum groups of 10 patients ensuring safe distancing (at least 2 m) and staggered invitations.

After identification of index case, more stringent measures were introduced, including “double-checking” of the COVID-19 symptom screening questionnaire by verbal interrogation, at each dialysis session. Patients were advised to immediately report COVID-19 symptoms should these occur on non-dialysis days. A document was handed to each patient indicating the adherence to preventative strategies, risk of dialysis closure, curtailment of social activity and details of dialysis Unit modification over the subsequent two-week period. The dialysis sessions were modified to two per day from Monday to Sunday (additional dialysis sessions were created on Sunday). The dialysis distance was increased to accommodate five patients rather than eight (dialysis distance approximately 2 m apart from the previous 1 m). All HCWs were instructed not to work at other dialysis units.

We considered the following biomarker values to be abnormal: NLR ≥ 4; D-Dimer ≥ 0.5 μg/ml; CRP ≥ 5 mg/L (specific laboratory recommendations for an abnormal value) (Lancet Laboratories, 2020). We assumed that individuals with abnormal biomarkers may be at a higher risk for COVID-19 and/or that these abnormal results may be associated with underlying undetected SARS-CoV-2 infection (false negative tests). We wished to test our assumption that abnormal biomarkers may be associated with false negative SARS-CoV-2 tests. Reports have suggested that biomarkers may be used to diagnose SARS-CoV-2. However, acute phase reactants may also be abnormal in individuals with chronic illnesses.

Therefore, Individuals with initial negative RT-PCR and abnormal biomarkers (one or more) were subjected to further scrutiny during the second week after the initial tests were performed, i.e. repeat RT-PCR and biomarkers (retest subgroup). The retest timeline was based on the assumption that “potential high infectivity” persisted for 2 weeks. Patients and HCWs were analyzed separately as we assumed that the risk of infectivity may be different. Some HCWs worked at more than one dialysis facility and may have been responsible for potential unit cross infection. We also anticipated that comorbidities may be less prevalent in HCWs.

Those with negative RT-PCR tests and normal biomarkers were not retested (non-retest subgroup). Prior to retesting, patients underwent segregated dialysis as a group, at the end of the allocated day, allowing for subsequent Unit disinfection. The HCWs remained in isolation until the retest results became available.

**FIGURE 1:**
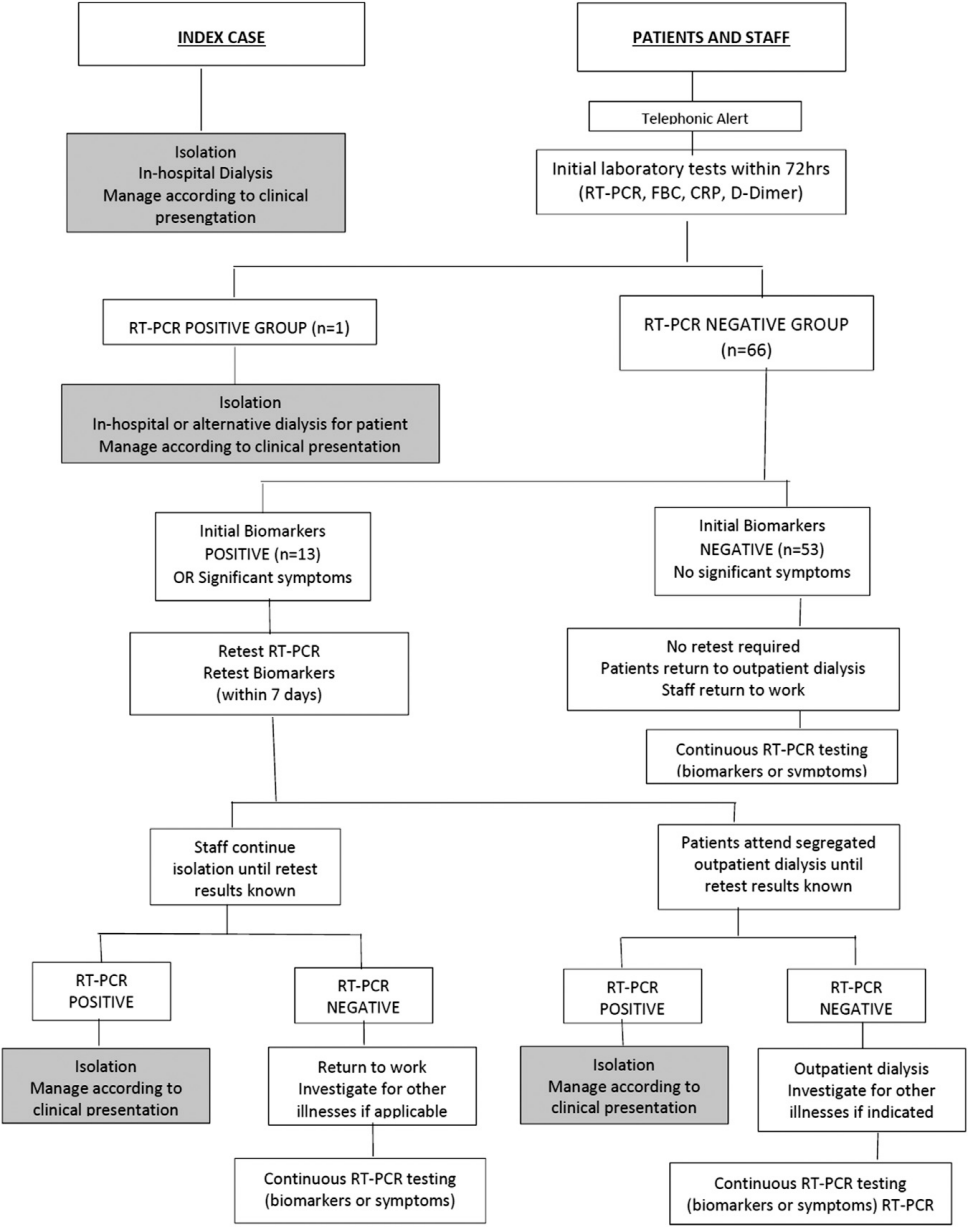
Clinical guideline implemented in the Unit for infection prevention and control.

A guideline was developed for the management of HCWs and patients as part of the infection control and prevention measures. Individuals were therefore categorized into: RT-PCR positive group, RT-PCR negative group, retest subgroup and non-retest subgroup (Figure 1).

### Data Collection and Analysis

All individuals filled a laboratory questionnaire which included demography, medical history and symptoms. A manual file was used to collect data for each individual. This included the questionnaire, consent form and original laboratory result sheets. Data was transcribed into an excel spread sheet for analysis. Continuous variables were expressed as the mean ± standard deviation (SD), if normally distributed or as the median (interquartile range (IQR)) in others; categorical variables were described as the count (%). All analyses were done with STATA version 15.

### Ethical Considerations

All individuals provided written consent and confidentiality was maintained by anonymizing the participants. The study was approved by the Sefako Makgatho Health Sciences University Research Ethics Committee (SMUREC/D/93/2020 (J).

## Results

Demographic and baseline characteristics of all 68 individuals are presented in Table 1. The RT-PCR positive index case (RT-PCR positive group, Figure 1) had normal biomarker values two months prior to the positive RT-PCR. The NLR was 2.2 and the CRP 4.2. Assessment of the initial and retested NLR and CRP showed a return to normal (Table 2). Although an increase in D-Dimer was noted in the index case and there were no clinical symptoms of COVID-19 over the 2 week period. He was therefore monitored as an outpatient. Two negative RT-PCR tests were obtained (two and three weeks respectively after initial diagnosis). He returned to outpatient dialysis uneventfully.

**TABLE 1:** Demographics and baseline characteristics of patients and HCWs.

Variables	Patients (*N* = 57)	HCWs (*N* = 11)
Gender	32 males; 25 females	2 males; 9 females
Age (yr) mean ±sd	52 ± 15	41.72 ± 4.56
Weight (kg) mean ±sd	73.98 ± 26.67	78.3 ± 15.19
Height(m) mean ±sd	1.63 ± 0.08	1.6 ± 0.07
BMI(kg/m^2^) mean ±sd	26 ± 10.35	31.01 ± 5.45
Employed	21	11
Smoker	5	2
HT	40	1
DM	17	0
CVS	20	1
Asthma	3	0
Malignancy	1	0

HT, hypertension; DM, diabetes mellitus; CVS, cardiovascular disease; BMI, body mass index.

**TABLE 2:** Biomarker comparison for patients and health care workers.

	Initial biomarkers			Retest biomarkers		
	NLR	D Dimer	CRP	NLR	D Dimer	CRP
Index case	4.69	0.55	23.1	2.65	2.65	4.7
2nd positive patient	24.29	0.32	182	6.86	—	40.3
RT-PCR negative group (biomarker positive)						
Patients						
1	4.41	1.26	8.5	3.8	1.75	11.4
2	2.33	4.31	2.3	3.8	1.45	1.8
3	3.1	6.34	1.5	n/a	n/a	n/a
4	11.15	0.8	<1.0	2.45	0.42	<1.0
5	6.68	1.52	7.3	1.63	0.68	1.3
6	2.6	9.13	60.6	2.09	7.39	43.6
7	0.98	9.41	8.4	0.79	8.54	15.3
8	1.85	1.28	20.3	0.95	1.5	9.3
9	4.73	1.78	17	1.33	0.74	2.8
10	4.42	1.41	4.6	1.7	n/a	5.5
HCWs						
1	1.76	0.6	18	1.68	0.47	11.4
2	0.69	0.52	7	0.47	0.37	4.3
3	1.17	0.72	31.1	1.14	1.08	30.1

We identified a second RT-PCR positive patient (RT-PCR positive group, Figure 1) from the initial tests. Within 48 h of being tested, the condition of this 74-year-old female (known IgG4 disease) deteriorated and she was admitted to hospital with a diagnosis of COVID-19 and septicaemia. At admission she presented with fever (38.5°C) and rigors. Clinical evaluation revealed a pulse rate of 98/min, BP of 114/85 mm of Hg, oxygen saturation of 90% and respiratory rate of 28/min. She was assessed as having moderate COVID-19 and her usual dialysis protocol was followed during hospital admission. No ventilation was required. Dynamic assessment of biomarkers revealed sequential improvement. This was congruent with clinical improvement and a negative RT-PCR was obtained prior to discharge on day 10 (Table 2).

The remainder of the patients and all HCW had negative RT-PCR tests during initial testing. In this negative RT-PCR group there were 66 individuals who were further classified into two subgroups based on abnormal (retest group) and normal (non-retest) biomarkers (Figure 1).

a. Retest subgroup (RT-PCR and Biomarkers were repeated in the second week): (*n* = 13: 10 patients and three HCWs). These individuals had no significant symptoms during initial testing. Although these individuals had abnormal initial biomarkers, none had positive RT-PCR during retesting, and none reported COVID-19 symptoms. There was no deterioration in median biomarker values between initial biomarkers [NLR 2.6 (IQR 1.7–4.4); D-Dimer 1.41 (IQR 0.8–4.3); CRP 8.4 (IQR 6.4–18.6)] and retest biomarkers [NLR 1.6 (IQR 1.1–2.1); D-Dimer 1.1 (IQR 0.6–1.6); CRP 7.4 (IQR 2.6–12.4)]. One of the HCWs was found to have a sustained high CRP and D-Dimer during initial testing and retesting. This was an incidental finding and the HCW was referred to a physician for further evaluation.

Non-Retest subgroup (return to dialysis without retesting): (*n* = 53: 45 patients and eight HCW) These individuals had normal initial biomarkers and did not have COVID-19 symptoms. They were not submitted to retesting. They returned to dialysis and were monitored by symptomatic screening. The HCW returned to work. Clinical and symptom assessment after 14 days did not reveal COVID-19 symptoms.

Abnormal CRP, NLR and D-Dimer did not identify individuals with potential false negative RT-PCR tests and generally there was no deterioration in biomarker values.

Tracking and tracing pursuits identified two positive SARS-CoV-2 family members of the second positive patient (same household). Community transmission was thought to be responsible for the SARS-CoV-2 infection in the index case. Subsequent interaction between the index case and second positive case may have occurred in the dialysis “waiting area.”

## Discussion

This study outlines the management of a group of dialysis dependent ESRF patients after one of them was tested positive for SARS-CoV-2. The Unit had already implemented SARS-CoV-2 preventative measures prior to identifying the index case. Avoidance of Unit closure was a priority. To counteract spread of infection within the Unit, the Unit performed early universal RT-PCR testing of all patients and HCWs. A second SARS-CoV-2 positive patient was identified and immediately isolated. During the subsequent 2-week period of “potential high infectivity,” we reinforced prevention policies, monitored COVID-19 symptoms, further decreased patient interaction during dialysis activity, advised against social interaction, monitored biomarkers and selectively repeated RT-PCR tests based on abnormal biomarkers. Selective retesting of RT-PCR, based on abnormal biomarkers did not reveal any other infected individuals. Generally, biomarkers did not deteriorate over the 2-week period.

The initial exposure and periods of infectivity in both positive patients were unknown. It is likely that pre-existing preventative measures may have played a role in limiting transmission of infection within the unit. However, once there is an outbreak relying only on symptom based isolation may be insufficient to safely prevent spread of infection (Basile et al., 2020). It is not unusual for ESRF patients to present with cough, malaise and tiredness. The other factors that need to be considered include, the immediate increased infective risk, asymptomatic transmission, incubation period, comorbidities and significant rates of false negative RT-PCR tests (Henry and Lippi, 2020).

Furthermore, the incidence of COVID-19 in urban dialysis patients have been reported to be 19.6%.9 The incidence of asymptomatic SARS-CoV-2 infection in an analysis of 65 dialysis facilities was reported to be 21.4% (Xiong et al., 2020). Immediate (and universal) RT- PCR testing seems logical and has been recommended by others (Wikramaratna et al., 2020).

Reiterating COVID-19 preventative strategies and highlighting risk of dialysis unit closure are important to mitigate spread of infection (Hu et al., 2020). Complacency, particularly in cluster and interactive environments are to be expected. Telephonic and written communication were both used to make patients and staff aware of the outbreak, modified dialysis sessions and need social distance vigilance.

We used abnormal biomarkers to objectively select a subset of individuals with a higher probability of underlying impactful illness or as predictors of undetected SARS-CoV-2 infection. (Cruz et al., 2011; Nakazato et al., 2015; LABMATE, 2020; Lancet Laboratories, 2020; Ferrari et al., 2020). In these individuals we considered the possibility of false negative RT-PCR tests and if infected, the risk of severe COVID-19. (Henry and Lippi, 2020; Wikramaratna et al., 2020; WHO. 2020b). They were therefore submitted to RT-PCR and biomarker retesting.

Improvement in biomarkers were noted in the asymptomatic index patient; the RT-PCR positive symptomatic patient and the retested subgroup (RT-PCR negative) (Table 2). The increase in D-Dimer in the index case was probably related to a thrombosed arterio-venous fistula previously used for haemodialysis.

In the RT-PCR positive patients the improved biomarker trend corresponded with the subsequent negative RT-PCR tests. This confirms the value of monitoring biomarker trends in COVID-19 (Chen et al., 2020b; Kermali et al., 2020; Lan et al., 2020; Terpos et al., 2020; Ulhaq and Soraya, 2020).

The abnormal biomarkers noted in the 10 RT-PCR negative patients (initial tests) could be explained by intrinsic comorbidities found in ESRF patients. There was almost uniform biomarker improvement on retesting. Patients 2, 4 and 9 in particular, showed improvement in biomarker values (Table 2). The reason for this improvement is unclear, but is likely related to improvement in general health or transient biomarker variation noted in ESRF patients. Abnormal biomarkers and biomarker variation in renal failure patients may render them as poor predictors of SARS-CoV-2 infection (Nakazato et al., 2015; Cruz et al., 2011). In this study, abnormal biomarkers were not predictive of SARS-CoV-2 infection on retesting. (Ferrari et al., 2020; LABMATE, 2020; Mardani et al., 2020). This warrants further investigation in a larger cohort of ESRF patients. Although the early implementation of preventative measures within the unit may have been crucial in limiting the spread of infection, it was the identification of the asymptomatic index patient that prompted universal RT-PCR testing. This also led to a heightened awareness of the risk of unit closure and prompted more stringent preventative strategies. Random or periodic universal RT-PCR testing in ESRF patients requires further evaluation.

### Limitations

This study is restricted to a single dialysis unit. The strategy used may, therefore, not be generalized to other units with limited RT-PCR test capability. We also made an assumption that the biomarker negative sub-group had a low risk of infection and therefore, these individuals were not retested. We could therefore have underestimated the rate of infection. A strategy of retesting all individuals needs further evaluation.

## Conclusion

Outpatient dialysis facilities are susceptible to outbreaks in the event of SARS-CoV-2 exposure. While routine preventative and mitigation strategies are imperative, a SARS-CoV-2 outbreak may necessitate additional proactive steps to counteract spread of infection.

This includes early RT-PCR testing of all patients and HCWs and creating further awareness of the risk of transmission and modifying preventative strategies. Abnormal biomarkers may be poorly predictive of SARS-CoV-2 infection in ESRF patients due to underlying illnesses. Observing dynamic changes in biomarkers in RT-PCR positive and negative-patients may provide insights into general state of health.

## Data Availability

The data analyzed in this study is subject to the following licenses/restrictions: None. Requests to access these datasets should be directed to jaypvascular@gmail.com.
